# Relationship between soil and species diversity in typical forest stands in Xianrendong, Liaoning Province based on plant-soil feedback

**DOI:** 10.1371/journal.pone.0306568

**Published:** 2024-07-05

**Authors:** Weixin Du, Hua Zhang

**Affiliations:** Department of Geographical Sciences, Liaoning Normal University, Dalian, Liaoning, China; Central South University of Forestry and Technology, CHINA

## Abstract

Exploring the relationship between soil properties and species diversity in typical forest stands in Liaoning Xianrendong National Nature Reserve will help maintain the stability of forest communities in the transition zone between flora in Changbai and North China. Based on the plant-soil feedback theory, community sample data from nine typical forest stands in the study area and experimental test data from 54 soil samples, we selected indexes of soil physical and chemical properties based on the minimum data set (temperature, compactness, capillary pore space, bulk weight, capillary water holding capacity, drainage capacity, soil water storage, conductivity, pH, organic matter, Ca, Fe, K, N and P). We adopt the research method of classical statistical analysis. The soil properties of nine typical stands in Xianrendong National Nature Reserve of Liaoning Province were systematically analyzed. The relationship between soil properties and forest stands’ species diversity was quantified using correlation and redundancy analyses. The Pearson correlation analysis results showed significant positive correlations between the Gleason abundance index (arbors) with conductivity, pH, organic matter, Ca, N and P; Pielou’s evenness index (arbors) with bulk weight and Fe. Significant negative correlations between the Gleason abundance index (arbors) with capillary pore space, bulk weight, drainage capacity, soil water storage and capillary water holding capacity; Simpson dominance index and Shannon–Wiener diversity index with capillary water holding capacity, drainage capacity and soil water storage; Pielou’s evenness index (arbors) with Ca and N. The natural moisture content and clay particles are neutral feedback. The results showed that the feedback mechanism of soil physicochemical properties on stand species diversity was complex, which was conducive to species coexistence and community stability.

## Introduction

Forest soils develop under forest vegetation. A combination of physical, chemical, and biological properties govern their characteristics and fertility, which are fundamental to ensuring species diversity, the sustainable development and stability of forest ecosystems, and the growth of forest communities [1].

Domestic and international studies on forest soil shape and species diversity have focused on the drivers of species diversity and the relationship between soil characteristics and species diversity. Forest soil properties are closely related to species diversity; for example, Appiah-Badu’s [2] study on the relationship between species diversity, physicochemical soil properties, and land use in the forests of Awudua, Ghana, found that land use practices in rubber plantations negatively impacted plant species diversity and soil properties. Zhou’s [3] study on the species diversity and physicochemical soil properties of evergreen broadleaf forest communities in the Bifeng Gorge area of Sichuan found that soil organic matter, total N, and soil water content were the main factors affecting community species diversity. Li [4] studied forest plant communities on the southern slope of Daiyun Mountain and found that the soil’s total P content was the driving factor behind phylogenetic diversity and species diversity. Onyekwelu’s [5] study on the species diversity and soil properties of primary and degraded rainforests in Southwestern Nigeria found that differences in soil properties could not be attributed to the effects of forest degradation.

Although some scholars have conducted preliminary studies on vegetation types and distribution, community structure and succession, and environmental effects in the Xianrendong National Nature Reserve of Liaoning Province [6–9], there is a lack of systematic research on the relationship between physicochemical soil properties and the species diversity of typical stands in protected areas.

The plant–soil feedback theory indicates that soil properties are important to the growth and development of forest vegetation [10]. Therefore, we selected Xianrendong National Nature Reserve in Liaoning Province as our research area. This area has been preserved without human interference and is located in the transition zone between North China and Changbai flora. It has a humid temperate monsoon climate, with marine influence. In order to construct an evaluation system, appropriate indexes related to experimental data were selected from nine typical zonal forest vegetation soils and their physical and chemical properties, and the soil physical and chemical characteristics of nine typical zonal forest vegetation types were evaluated.

In this study, we reveal the feedback relationship between soil traits and species diversity of typical forest communities in the Changbai, North China, plant transition zone. We also provide a scientific basis for protecting forest vegetation in Xianrendong National Nature Reserve and basic data for studying forest nutrient cycling mechanisms in the Changbai, North China, plant transition zone.

## Materials and methods

### 2.1 Overview of the study area

**Fig 1:**
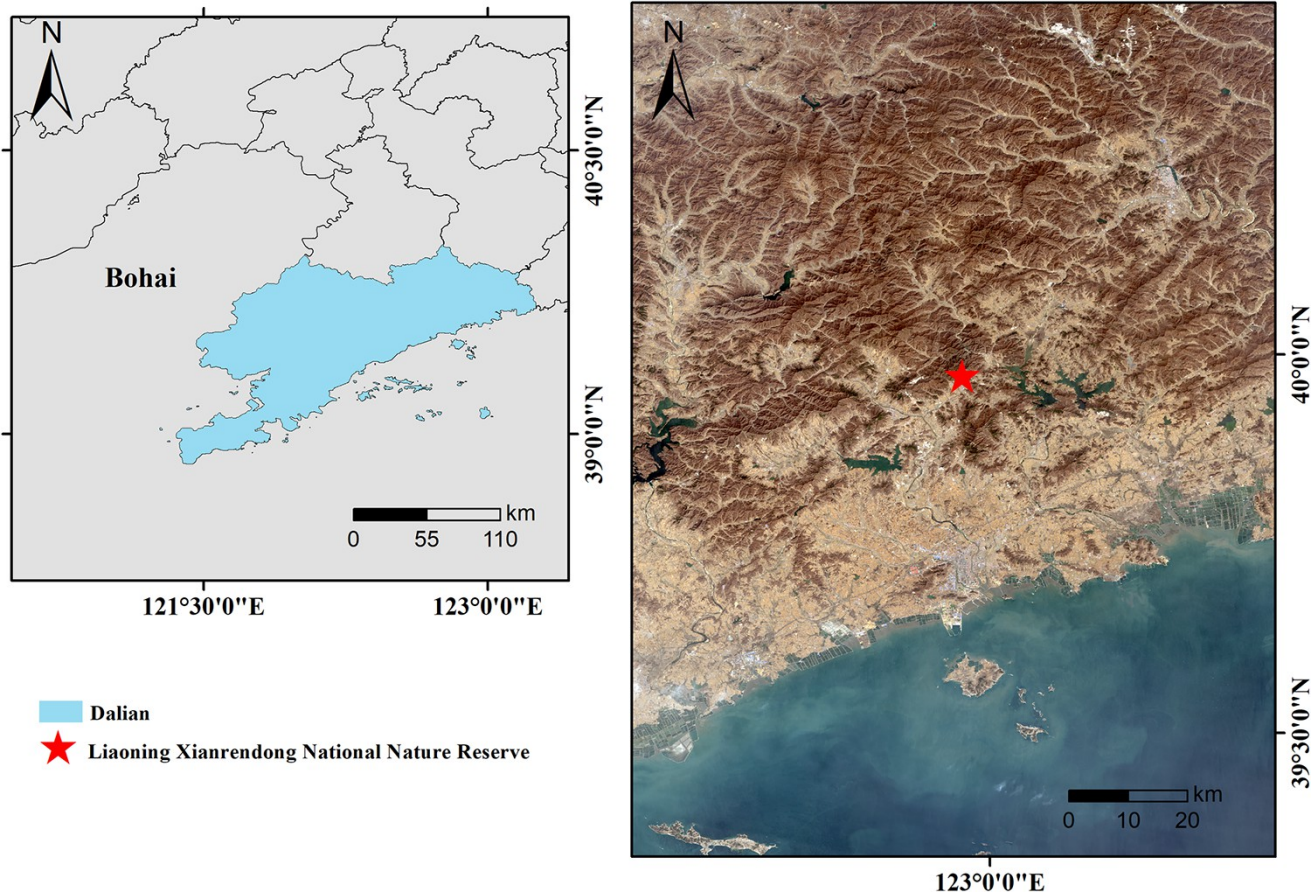
Location map of the study area. **Note:** Review drawing No: GS(2019)1682, Supervised by Ministry of Natural Resources of China.

Liaoning Xianrendong National Nature Reserve is located in the northern mountainous area of Zhuanghe, Dalian City, Liaodong Peninsula, bordering the Yellow Sea in the south and Qianshan Mountain in the north, with geographical coordinates of 122°53′24″~123°03′30″E and 39°54′00″~40°03′00″N. The reserve’s total area is 35.75 km^2^, 7.81 km^2^ of which is the core area, whose original vegetation is most protected. The buffer area was 8.76 km^2^, and the experimental area was 19.18 km^2^ (Fig 1). The reserve belongs to the hilly mountainous area of the Liaodong Peninsula, the southern extension of the Qianshan Mountain Range. It is located in the humid temperate monsoon climate zone, with some maritime climate characteristics. It is hot and humid in summer and cold and dry in winter, with an average annual temperature of 9.7°C, a frost-free period of 182 d, and an average annual precipitation of 974.6 mm. The soil types are typical quartzite brown loam and quartzite brown loamy soil [11]. *Pinus densiflora* coniferous forests, *Pinus densiflora*—*Quercus* coniferous mixed forests, and other zonal vegetation are the main conservation concerns in this area. They are all considered natural secondary forests.

### 2.2 Selection of sample site, collection and determination of soil samples

In Jun 2018, nine typical forest stands were selected for community sample plot surveys (sample plot area 20 × 30 m) in the core area of Liaoning Xianrendong National Nature Reserve according to forest growth characteristics. In each sample plot, three soil sampling points were set up along a diagonal line, totaling 27 sampling points. Each sampling point was divided into a 0–20 cm soil layer (hereinafter referred to as Layer A) and a 20–40 cm soil layer (hereinafter referred to as Layer B), and a total of 54 soil samples were collected. At the same time as sampling, Using the British Wet portable soil moisture temperature conductivity speed tester (Burwell, Cambridge, CB25 0EJ, UK), the soil temperature, conductivity and soil natural water content of Layer A was determined (re-peated five times). We measured the soil compactness of Layer A using an ST-6101 soil compactness meter (Spectrum Technologies, Aurora, IL, USA) (repeated five times). An Ezobel T2 GIS collector (Nanjing, Jiangsu, CHN) and handheld compass meter (Harbin, Heilongjiang, CHN) were used to collect basic information for each sample plot (Table 1).

**Table 1:** Basic information about the plot.

Stand number	Longitude (E)	Latitude (N)	Elevation (m)	Slope (°)	Aspect (°)	Slope	Soil layer thickness (cm)	Depression closure	Naming of typical forest stands and communities
1	122°57′54.24″	39°58′55.97″	176.6	14	198	Lower	80	0.70	*Pinus densiflora*—*Zanthoxylum schinifoilum*—*Carex callitrichos* var. *nana* community
2	122°56′19.13″	39°59′24.46″	231.8	26	230	Lower	110	0.85	*Quercus mongolica* + *Pinus densiflora*—*Rubus rataegifolius* -*Carex callitrichos* var. *nana* community
3	122°56′18.07″	39°59′22.57″	229.8	26	278	Lower	120	0.89	*Quercus mongolica*—*Rubus crataegifolius* -*Carex allitrichos* var. *nana* community
4	122°56′15.62″	39°59′19.46″	243.4	21	308	Lower	100	0.68	*Pinus densiflora*—*Rubus crataegifolius* + *Zanthoxylum schinifoilum* -*Carex callitrichos* var. *nana* community
5	122°56′05.33″	39°59′42.82″	239.3	19	295	Lower	110	0.65	*Pinus densiflora*—*Hododendron micranthum* + *Zanthoxylum schinifoilum* + *Symplocos paniculata* -*Carex callitrichos* var. *nana* community
6	122°57′33.68″	39°59′09.12″	161.9	30	113	Lower	50	0.75	*Pinus densiflora* + *Quercus acutissima*—*Parthenocissus tricuspidata* + *Zanthoxylum schinifoilum*—*Amphicarpaea edgeworthii* + *Cardamine leucantha* + *Arisaema angustatum* var. *peninsulae* community
7	122°57′46.02″	39°59′01.47″	175.0	25	218	Lower middle	120	0.75	*Quercus dentata* + *Quercus acutissima* + *Quercus mongolica*—*Indigofera kirilowii* + *Corylus heterophylla*—*Artemisia keiskeana* +*Carex callitrichos* var. *nana*+ *Achnatherum pekinense* community
8	122°57′58.18″	39°59′02.56″	211.5	27	219	Lower middle	90	0.65	*Quercus acutissima* + *Quercus variabilis—Zanthoxylum schinifoilum* + *Corylus heterophylla* -*Carex callitrichos* var. *nana* community
9	122°57′58.63″	39°58′58.67″	201.8	31	220	Lower middle	100	0.70	*Pinus densiflora* + *Quercus dentata* + *Quercus mongolica*—*Corylus heterophylla* -*Carex callitrichos* var. *nana* community

The collected soil samples were brought back to the laboratory to be naturally air-dried, crushed, removal of impurities (roots, stones, etc.), and sieved for testing.

1. Soil physical characteristics testing: About 5 g of soil samples with a 2-mm sieve were obtained from the prepared soil samples for pre-treatment. The soil particle size was tested using a Beckman LS13 320 (Beckman Coulter Inc., CA, USA) laser diffraction particle size analyzer and a particle size classification was performed using the American system of soil particle size classification standard [1]. Soil bulk weight, total porosity, capillary pore space, capillary water holding capacity, soil water storage capacity, and drainage capacity were determined using the ring knife method, with reference to the *Determination of moisture-physical properties of forest soils* [12].
2. Soil chemical characteristics test: 25 g of air-dried soil samples collected through a 1-mm sieve were obtained from the prepared soil samples. The pH value of the soil was determined using an ExStik series pH 100 waterproof pen pH value meter (Shanghai Sanxin Instrument Factory, Shanghai, China). The air-dried soil samples collected through 0.25-mm sieves weighed about 0.3 g, and the organic matter was determined by the potassium dichromate–sulfuric acid oxidation method. Soil samples collected through a 2-mm sieve weighed about 10 g. We used an SM-1 vibration grinder (Dandongbeiyuan Instrument Equipment Co. Ltd., Liaoning Province, China) to grind the soil samples, followed by BP-1. The silt press pressed the sample and delivered it to the Japanese Rigaku ZSX Primus II type automatic scanning X-ray fluorescence spectrometer (Rigaku, Shibuya, Tokyo) to determine the soil composition, especially the six nutrient elements necessary for plant growth. These six nutrients are nitrogen (N), phosphorus (P), potassium (K), calcium (Ca), magnesium (Mg), and iron (Fe) (Zhu et al. 2010).

### 2.3 Community species diversity measurement in forest stands

Based on the community science survey factors for the arbor, shrub, and herbaceous layers of each stand (arbor layer evaluated for the plant species name, number of plants, diameter at breast height, crown width and height; shrub layer evaluated for cover, plant species name, density, basal diameter, crown width and height; herbaceous layer evaluated for plant species name, cover, sub-cover, density, height and frequency). We used the Gleason abundance index, Shannon–Wiener diversity index, Simpson dominance index, and Pielou’s evenness index to characterize the diversity of community species in different forest stands. The calculation formula is [13]:

$$D_{G}=S/\mathrm{In}A$$ (1)

$$H=-\sum_{i=1}^{S}P_{i}\mathrm{In}P_{i}$$ (2)

$$D=1-\sum_{i=1}^{S}P_{i}^{2}$$ (3)

J=H/InS (4)

In the above formula, *D*_G_ is the Gleason abundance index, *A* is the area under investigation, and *S* is the plant species within the area under investigation. *H* is Shannon–Wiener diversity index, *P*_*i*_ is the important value of each plant species in arbor layer, shrub layer and herbaceous layer of forest community, and *s* is the number of plant species in arbor layer, shrub layer and herbaceous layer of forest community. *D* is Simpson dominance index and *J* is Pielou’s evenness index.

### 2.4 Statistical analysis of data

The variability of physicochemical characteristics in soil from the same soil layer in different stands is characterized by the coefficient of variation (CV), with coefficient of variation (%) = (standard deviation of test factors / mean of test factors) × 100. Generally, CV ≤ 10% is weak variability, 10% < CV < 100% is moderate variability, and CV ≥ 100% is strong variability [14].

Correlations between soil characteristics and stand species diversity in a typical stand were explored using Pearson’s correlation coefficient in SPSS 19.0 (IBM Corporation, Armonk, NY, USA) in Canoco 5.0 (Microcomputer Power, Ithaca, NY, USA), respectively.

## Results

### 3.1 Comprehensive soil properties of typical forest stands

#### 3.1.1 Soil physical properties

Table 2 shows the physical characteristics of soil in Layer A of a typical forest stand in Liaoning Xianrendong. The soil temperature of a typical forest stand is between 18.7 and 25.4°C, compactness is between 11.8 and 20.6 kg/cm^2^, bulk weight is between 0.92 and 1.48 g/cm^3^, total porosity is between 42.3% and 53.7%, capillary pore space is between 25.5% and 36.9%, natural water content is between 10.4% and 19.1%, soil water storage ranged from 107.9 to 311.0 mm, capillary water holding capacity ranged from 155.3 to 437.6 mm, and drainage capacity ranged from 136.8 to 433.2 mm. The coefficients of variation for soil temperature and total porosity were 8.6% and 7.2%, respectively, which indicated weak variability. The coefficients of variation for the remaining soil physical characteristics were 10.1%~33.9%, which indicated moderate variability. Although the soil temperature and total porosity did not differ significantly among the nine stands, the rest of the soil physical properties showed significant differences. It may be affected by soil parent material, topographic factors and litter content of different vegetation. Soil physical characteristics can directly affect the growth and development of plant roots and their absorption of nutrients and water, which is an important index to evaluate the basic soil environment and the water conservation function of stand.

**Table 2:** Physical characteristics of soil in Layer A of a typical forest stand in Xianrendong, Liaoning Province.

Stand number	Temperature (°C)	Compactness (kg/cm^2^)	Bulk weight (g/cm^3^)	Total porosity (%)	Capillary pore space (%)	Natural water content (%)	Soil water storage (mm)	Capillary water holding capacity (mm)	Drainage capacity (mm)
1	25.4±1.6	11.8±3.9	1.10±0.05	53.7±1.8	31.7±2.1	13.0±2.5	141.7±25.7	253.3±17.0	259.9±20.3
2	21.0±0.8	16.9±3.0	1.38±0.06	46.7±2.9	36.9±4.4	19.1±5.2	299.6±19.0	405.5±47.9	242.4±26.0
3	23.9±1.1	20.6±4.6	1.23±0.09	51.9±1.9	36.5±3.0	15.0±2.3	300.8±43.8	437.6±36.5	344.2±39.6
4	23.9±0.9	15.4±1.6	1.16±0.03	51.3±1.8	36.4±3.7	15.0±4.1	242.8±14.6	364.3±37.4	284.7±16.7
5	18.7±1.1	16.5±2.4	0.92±0.05	44.4±4.5	25.5±6.5	15.3±2.1	259.2±9.2	280.9±71.4	304.0±58.6
6	22.1±1.3	18.7±4.2	1.12±0.04	48.8±5.7	31.1±8.6	16.4±4.1	107.9±28.0	155.3±42.8	136.8±71.9
7	23.9±1.6	18.9±3.7	1.48±0.12	42.3±0.5	33.6±1.1	15.1±3.4	311.0±15.4	403.2±12.8	241.6±3.6
8	21.4±0.9	20.1±2.3	1.36±0.08	47.1±2.6	34.3±3.3	14.8±2.3	234.3±28.3	308.7±29.9	204.3±2.8
9	21.4±0.2	16.5±4.0	1.18±0.02	47.6±5.0	33.1±6.8	10.4±1.1	119.8±22.8	331.0±68.1	342.7±37.5
Mean±SD	22.4±1.9	17.3±2.6	1.21±0.16	48.2±3.4	33.2±3.4	14.9±2.2	224.1±76.0	326.6±83.7	262.3±62.7
CV (%)	8.6	14.8	13.2	7.2	10.1	14.8	33.9	25.6	23.9

**Note:** The data in this table are Mean±SD.

Table 3 shows the physical soil characteristics of Layers A and B in typical forest stands in Liaoning Xianrendong. The soil sand grains in Layer A of typical forest stands ranged from 9.0% to 33.0%, the silt grains ranged from 60.5% to 83.7%, and the clay grains ranged from 6.5% to 8.4%. The soil sand grains in Layer B ranged from 11.1% to 30.5%, the silt grains ranged from 63.8% to 81.2%, and the clay grains ranged from 5.7% to 7.7%. The coefficients of variation for soil sand grains and the remaining physical properties in Layers A and B were weak at 6.8%~8.8% These findings reflect the variation of soil sand grains in the nine forest stands, and that the rest of the soil’s physical characteristics have insignificant differences. The soil of Liaoning Xianrendong is characterized by sandy loam with chalk according to mean values of sand, chalk, and clay grains referenced in American soil classification standards.

**Table 3:** Physical characteristics of soil in Layers A and B of a typical forest stand in Xianrendong, Liaoning Province.

Stand number	Sand grains (2~0.05mm)(%)		Silt grains (0.05~0.002mm)(%)		Clay grains (<0.002mm)(%)	
	Layer-A	Layer-B	Layer-A	Layer-B	Layer-A	Layer-B
1	19.9±4.6	25.7±6.1	72.2±3.6	68.2±5.4	6.9±0.4	6.1±0.7
2	17.9±5.1	24.2±6.5	74.9±4.8	69.3±6.0	7.3±0.3	6.5±0.6
3	12.8±3.5	12.9±4.9	79.7±3.2	80.0±4.2	7.5±0.5	7.2±0.9
4	11.4±1.1	12.4±2.3	81.1±0.8	80.3±1.9	7.5±0.4	7.3±0.4
5	9.1±0.7	19.7±16.5	83.2±0.8	73.6±15.3	7.6±0.2	6.7±1.4
6	9.0±0.1	11.1±8.3	83.7±0.1	81.2±6.5	7.3±0.2	7.7±1.8
7	13.7±3.0	23.5±11.7	77.9±2.9	69.7±9.9	8.4±0.4	6.8±1.8
8	13.3±2.8	18.8±1.5	78.9±2.6	74.2±1.3	7.8±0.4	7.0±0.3
9	33.0±11.0	30.5±3.5	60.5±9.8	63.8±3.2	6.5±1.2	5.7±1.1
Mean±SD	15.6±7.0	19.9±6.3	76.9±6.8	73.3±5.7	7.4±0.5	6.8±0.6
CV (%)	45.3	31.9	8.8	7.7	6.8	8.6

**Note:** Layer A is the 0–20 cm soil layer and Layer B is the 20–40 cm soil layer. The data in this table are Mean±SD.

#### 3.1.2 Soil chemical properties

Tables 4 and 5 shows the soil’s chemical characteristics in Layers A and B of a typical forest stand in Xianrendong, Liaoning Province. The soil conductivity of Layer A in a typical forest stand ranges from 25.73 to 62.33 ms/m, pH values range from 4.88 to 5.46, organic matter ranges from 2.85% to 9.48%, Ca ranges from 2.566 to 7.808, Fe ranges from 42.333 to 102.202, K ranges from 16.606 to 26.485, Mg ranges from 6.995 to 9.490, N ranges from 3.437 to 7.854, P ranges from 0.017 to 0.977. Layer B’s soil pH ranges from 4.88 to 5.41, organic matter ranges from 2.32% to 7.30%, Ca ranges from 1.764 to 5.223, Fe ranges from 39.397 to 101.337, K ranges from 16.928 to 26.989, Mg ranges from 7.035 to 12.890, N ranges from 3.736 to 6.647, P ranges from 0.246 to 0.777. The coefficients of variation for soil pH and Mg in Layer A and soil pH in Layer B showed weak variation at 3.4%, 8.9%, and 3.4%, respectively. The coefficients of variation for the remaining chemical characteristics of soils in Layers A and B were moderate and ranged from 13.4% to 47.0%, respectively. These findings indicate that differences in the nine stands’ soil pH and Mg in Layer A and soil pH in Layer B are not obvious. By contrast, the remaining chemical characteristics of soils in Layers A and B are obvious differences. It may be affected by topographic factors and humus content. Soil chemical characteristics can reflect the level of soil nutrient content and affect and control the health status of plants, which is an important factor affecting forest productivity.

**Table 4:** Soil chemical characteristics of Layers A and B in a typical forest stand in Xianrendong, Liaoning Province.

Stand number	Conductivity (ms/m)		pH		Organic matter (%)	
	layer-a	layer-b	layer-a	layer-b	layer-a	layer-b
1	42.33±6.04	-	4.92±0.19	5.08±0.07	3.74±0.71	2.32±0.29
2	38.80±8.67	-	4.90±0.13	4.84±0.11	2.97±1.04	2.40±1.32
3	25.73±6.02	-	4.91±0.23	5.19±0.23	4.21±1.05	4.19±1.05
4	42.93±7.22	-	5.06±0.02	5.20±0.11	5.14±0.44	4.12±0.43
5	30.60±8.15	-	4.96±0.23	5.41±0.58	7.81±1.07	5.05±1.34
6	62.33±26.69	-	5.46±0.06	5.21±0.04	9.48±0.80	7.30±0.64
7	28.73±5.61	-	4.95±0.07	4.95±0.05	3.51±0.40	2.42±0.49
8	32.27±3.47	-	4.88±0.15	5.10±0.08	5.32±0.42	3.13±0.46
9	36.67±9.36	-	4.93±0.18	4.88±0.09	2.85±0.05	2.50±0.32
Mean±SD	37.82±10.33	-	5.00±0.17	5.10±0.17	5.00±2.14	3.71±1.57
CV (%)	27	-	3.4	3.4	43	42

**Note:** Layer A is the 0–20 cm soil layer and Layer B is the 20–40 cm soil layer. The data in this table are Mean±SD.

**Table 5:** Soil chemical characteristics of Layers A and B in a typical forest stand in Xianrendong, Liaoning Province.

Stand number	Ca		Fe		K		Mg		N		P	
	layer-a	layer-b	layer-a	layer-b	layer-a	layer-b	layer-a	layer-b	layer-a	layer-b	layer-a	layer-b
1	4.656± 0.366	3.447± 0.586	48.634± 2.159	52.070± 2.345	23.953± 0.935	24.420± 0.932	7.809± 0.491	8.265± 0.407	4.968± 0.570	4.802± 0.274	0.386± 0.044	0.308± 0.018
2	3.618± 1.024	2.848± 0.882	48.979± 1.558	50.380± 0.466	23.730± 2.198	24.271± 2.733	8.094± 0.586	8.694± 0.557	4.632± 0.511	4.765± 0.660	0.266± 0.044	0.235± 0.033
3	5.093± 0.388	5.223± 0.342	46.018± 1.146	47.593± 2.857	20.034± 1.449	19.565± 1.630	7.473± 0.083	7.669± 0.266	5.571± 0.242	5.164± 0.777	0.323± 0.019	0.331± 0.031
4	5.651± 0.786	5.189± 0.570	43.562± 1.324	44.285± 0.475	20.766± 0.476	21.181± 0.275	7.870± 0.520	8.202± 0.275	6.130± 0.297	5.104± 0.311	0.432± 0.019	0.356± 0.047
5	4.817± 0.531	4.255± 0.553	44.494± 1.032	42.360± 10.801	19.800± 0.091	18.897± 3.290	7.791± 0.233	7.311± 1.389	7.099± 0.519	5.450± 0.965	0.510± 0.031	0.502± 0.152
6	7.808± 0.627	4.833± 0.836	42.333± 0.150	39.397± 3.320	19.720± 0.900	18.361± 1.739	7.105± 0.619	7.035± 0.151	7.854± 0.291	6.647± 0.851	0.977± 0.002	0.777± 0.259
7	4.209± 0.316	2.939± 0.512	43.096± 0.486	48.701± 3.106	26.485± 0.746	26.989± 0.764	6.995± 0.124	7.805± 0.858	4.729± 0.327	4.472± 0.195	0.017± 0.017	0.286± 0.015
8	4.987± 0.171	3.823± 0.344	44.007± 0.411	46.235± 1.854	24.422± 0.745	25.118± 1.749	7.394± 0.392	7.605± 0.681	5.981± 0.186	4.958± 0.574	0.501± 0.002	0.381± 0.029
9	2.566± 0.135	1.764± 0.470	102.202± 34.942	101.337± 54.990	16.606± 4.865	16.928± 8.130	9.490± 2.786	12.890± 8.547	3.437± 0.326	3.736± 0.292	0.240± 0.015	0.246± 0.060
Mean ±SD	4.823± 1.360	3.813± 1.114	51.481± 18.068	52.484± 17.668	21.724± 2.918	21.748± 3.337	7.780± 0.695	8.386± 1.662	5.600± 1.271	5.011± 0.740	0.444± 0.208	0.380± 0.159
CV (%)	28	29	35	34	13	15	8.9	20	23	15	47	42

**Note:** Layer A is the 0–20 cm soil layer and Layer B is the 20–40 cm soil layer. The data in this table are Mean±SD.

### 3.2 Plant species diversity in typical forest stands

The Gleason abundance index, Shannon–Wiener diversity index, Simpson dominance index, and Pielou’s evenness index for the arbor layers in a typical forest stand in Xianrendong, Liaoning Province, ranged from 0.003 to 0.012 species/m^2^, 0.344~1.389, 0.046 to 0.721, and 0.161 to 0.990, respectively. The mean values of the Gleason abundance index, Shannon–Wiener diversity index, Simpson dominance index, and Pielou’s evenness index were (0.007±0.003) species/m^2^, 0.828±0.394, 0.458±0.216, and 0.639±0.279, with coefficients of variation of 43.0%, 47.6%, 47.3%, and 43.7%, respectively. The Gleason abundance index, Shannon–Wiener diversity index, Simpson dominance index, and Pielou’s evenness index for shrub layers ranged from 0.072 to 0.224 species/m^2^, 0.825 to 1.939, 0.457 to 0.829, and 0.741 to 0.922, respectively. The mean values of the Gleason abundance index, Shannon–Wiener diversity index, Simpson dominance index, and Pielou’s evenness index were (0.117 ± 0.041) species/m^2^, 1.487 ± 0.317, 0.695 ± 0.103, and 0.835 ± 0.060, with coefficients of variation of 35.4%, 21.3%, 14.8%, and 7.1%, respectively. The Gleason abundance index, Shannon–Wiener diversity index, Simpson dominance index, and Pielou’s evenness index for herbaceous layers ranged from 2.800 to 5.600 species/m^2^, 1.343 to 1.948, 0.650 to 0.819, and 0.720 to 0.937. The mean values of the Gleason abundance index, Shannon–Wiener diversity index, Simpson dominance index, and Pielou’s evenness index were (4.111 ± 0.875) species/m^2^, 1.573 ± 0.167, 0.726 ± 0.042, and 0.838 ± 0.061, with coefficients of variation of 21.3%, 10.6%, 5.8%, and 7.2%, respectively. The coefficients of variation for Pielou’s evenness index for the shrub layer and the Simpson dominance index and Pielou’s evenness index for the herbaceous layer had weak variation at 5.8%~7.2%. The coefficients of variation for species diversity index in the rest of the stands has moderate variation at 10.6%~47.6%, respectively.

**Fig 2:**
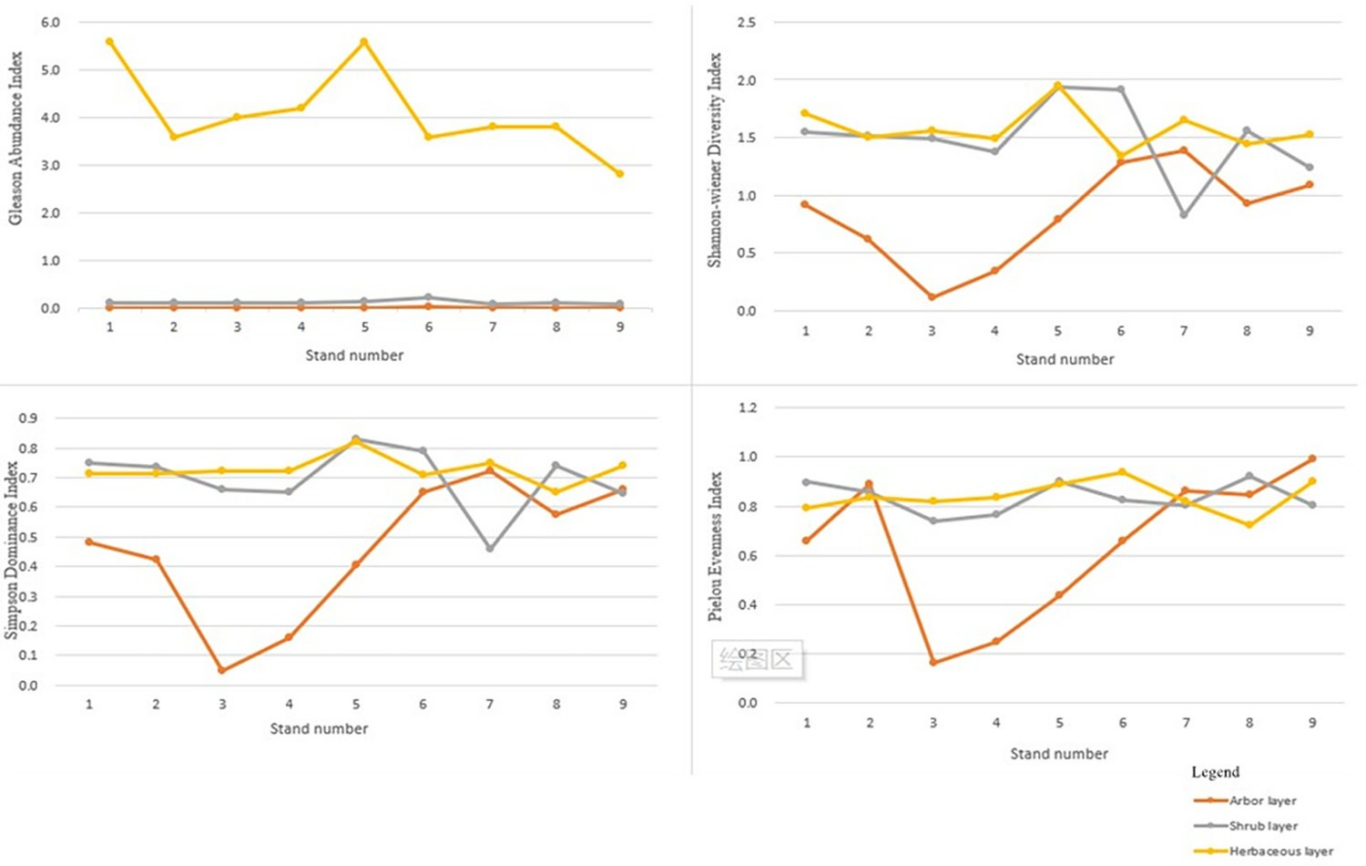
Folding line of species diversity index of typical forest stands in Xianrendong, Liaoning Province.

Fig 2 shows the species diversity indices of typical forest stands in Liaoning Xianrendong. The Gleason abundance index of nine typical forest stands is herbaceous layer > shrub layer > arbor layer; the Shannon–Wiener diversity index is herbaceous layer > shrub layer > arbor layer, except for *Quercus dentata* + *Quercus acutissima* + *Quercus mongolica*—*Indigofera kirilowii* + *Corylus heterophylla*—*Artemisia keiskeana* + *Carex callitrichos* var. *nana* + *Achnatherum pekinense* community, which is herbaceous layer > arbor layer > shrub layer, *Quercus mongolica* + *Pinus densiflora*—*Rubus rataegifolius*—*Carex callitrichos* var. *nana* community, *Pinus densiflora* + *Quercus acutissima*—*Parthenocissus tricuspidata* + *Zanthoxylum schinifoilum*—*Amphicarpaea edgeworthii* + *Cardamine leucantha* + *Arisaema angustatum* var. *peninsulae* community and *Quercus acutissima* + *Quercus variabilis*—*Zan-thoxylum schinifoilum* + *Corylus heterophylla*—*Carex callitrichos* var. *nana* community for shrub layer > herbaceous layer > arbor layer; Simpson dominance index of shrub layer > herbaceous layer > arbor layer, except for *Quercus mongolica*—*Rubus crataegifolius*—*Carex allitrichos* var. *nana* community and *Pinus densiflora*—*Rubus crataegifolius* + *Zanthoxylum schinifoilum*—*Carex callitrichos* var. *nana* community for herbaceous layer > shrub layer > arbor layer, *Quercus dentata* + *Quercus acutissima* + *Quercus mongolica*—*Indigofera kirilowii* + *Corylus heterophylla*—*Artemisia keiskeana* + *Carex callitrichos* var. *nana* + *Achnatherum pekinense* community and *Pinus densiflora* + *Quercus dentata* + *Quercus mongolica*—*Corylus heterophylla*—*Carex callitrichos* var. *nana* community for herbaceous layer > arbor layer > shrub layer. The Pielou’s evenness index was higher in the arbor, shrub, and herbaceous layers of nine stands, and lower in the *Quercus mongolica*—*Rubus crataegifolius*—*Carex allitrichos* var. *nana* community, *Pinus densiflora*—*Rubus crataegifolius* + *Zanthoxylum schinifoilum*—*Carex callitrichos* var. *nana* community, and *Pinus densiflora*—*Hododendron micranthum* + *Zanthoxylum schinifoilum* + *Symplocos paniculata*—*Carex callitrichos* var. *nana* community arbor layers.

In summary, the Gleason abundance index (arbors, shrubs), and Pielou’s evenness index were higher in the *Pinus densiflora* + *Quercus acutissima*—*Parthenocissus tricuspidata* + *Zanthoxylum schinifoilum*—*Amphicarpaea edgeworthii* + *Cardamine leucantha* + *Arisaema angustatum* var. *peninsulae* community. The Shannon–Wiener diversity index (shrubs, herbaceous) and Simpson dominance index (shrubs, herbaceous) were higher in the *Pinus densiflora*—*Hododendron micranthum* + *Zanthoxylum schinifoilum* + *Symplocos panicu-lata*—*Carex callitrichos* var. *nana* community.

### 3.3 Relationship between soil properties and species diversity in forest stands

#### 3.3.1 Pearson correlation between soil properties and species diversity in forest stands

**Fig 3:**
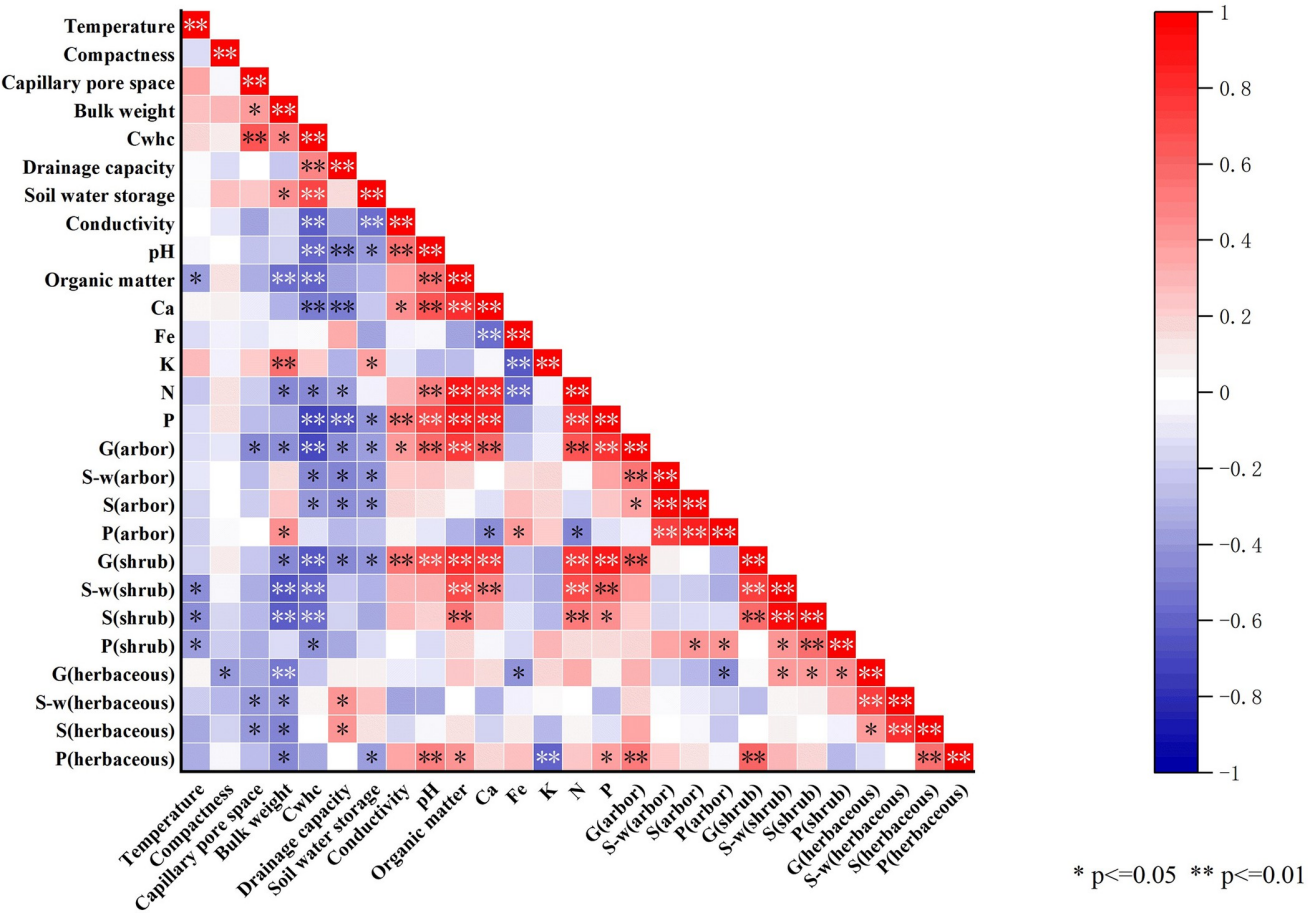
Pearson correlation coefficients between soil properties and species diversity indices in a typical forest stand in Xianrendong, Liaoning. **Note:** Cwhe, Capillary water holding capacity; G, Gleason abundance index; S-w, Shannon-wiener diversity index; S, Simpson dominance index; P, Pielou evenness index.

Fig 3 shows the Pearson’s correlation coefficients between soil properties and stand species diversity indices in Layer A of Liaoning Xianrendong forest stands. It also shows a close relationship between the values of the soil properties of typical forest stands and the stand species diversity index. The Gleason abundance index (arbors) with capillary pore space, bulk weight, drainage capacity and soil water storage; Shannon–Wiener diversity index (arbors) and Simpson dominance index (arbors) with capillary water holding capacity, drainage capacity and soil water storage; Pielou’s evenness index (arbors) with Ca and N; Gleason abundance index (shrubs) with bulk weight, drainage capacity and soil water storage; Shannon–Wiener diversity index (shrubs) and Simpson dominance index (shrubs) with temperature; Pielou’s evenness index (shrubs) with temperature and capillary water holding capacity; Gleason abundance index (herbaceous) with compactness and Fe; Shannon–Wiener diversity index (herbaceous) and Simpson dominance index (herbaceous) with capillary pore space and bulk weight; Pielou’s evenness index (herbaceous) was significant negative correlations with bulk weight and soil water storage (p<0.05).

The Gleason abundance index (arbors and shrubs) with capillary water holding capacity; Shannon–Wiener diversity index (shrubs) and Simpson dominance index (shrubs) with bulk weight and capillary water holding capacity; Gleason abundance index (herbaceous) with bulk weight; Pielou’s evenness index (herbaceous) was negative correlations with K (p<0.01).

The Gleason abundance index (arbors) with conductivity; Pielou’s evenness index (arbors) with bulk weight and Fe; Simpson dominance index (arbors) with P; Shannon–Wiener diversity index (herbaceous) and Simpson dominance index (herbaceous) with drainage capacity; Pielou’s evenness index (herbaceous) was significant positive correlations with organic matter and P (p<0.05).

The Gleason abundance index (arbors) with pH, organic matter, Ca, N and P; Gleason abundance index (shrubs) with conductivity, pH, organic matter, Ca, N and P, Shannon–Wiener diversity index (shrubs) with organic matter, Ca, N and P; Simpson dominance index (shrubs) with organic matter and N; Pielou’s evenness index (herbaceous) was positive correlations with pH(p<0.01).

The results showed that the relationship between soil physical and chemical properties and species diversity was complex, with positive feedback, negative feedback and neutral feedback, and this complex feedback relationship was conducive to the maintenance of stand species diversity.

#### 3.3.2 Redundancy analysis of physicochemical soil characteristics and species diversity in forest stands

**Fig 4:**
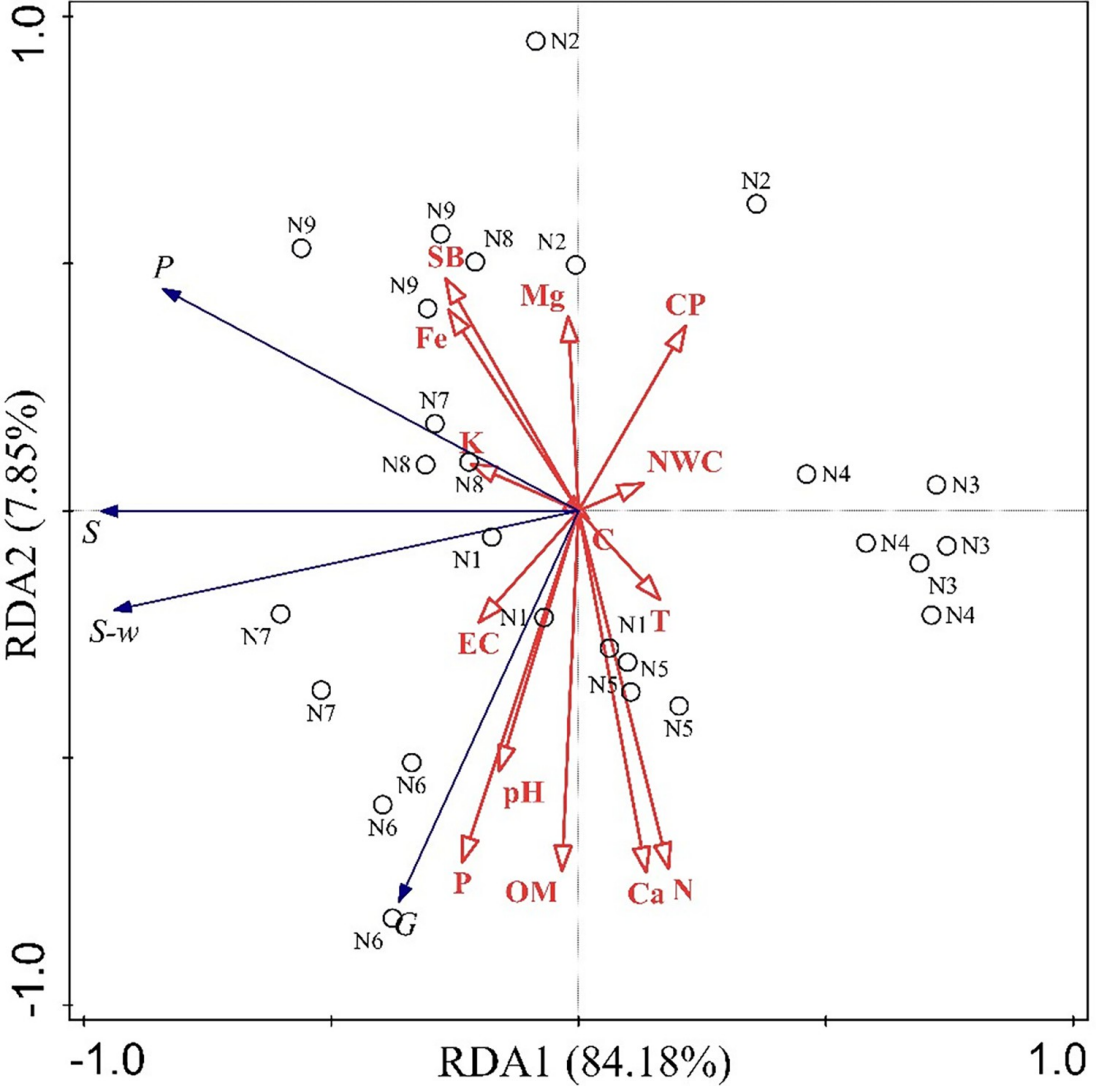
Redundancy analysis between soil physical and chemical characteristics and species diversity in a typical forest stand in Xianrendong, Liaoning Province. **Note:** OM, Organic matter; T, Temperature; CP, Capillary pore space; C, Compactness; EC, Conductivity; NWC, Natural water content; SB, Bulk weight.

Table 6 shows the redundancy analysis ranking between physicochemical soil characteristics and stand species diversity in Layer A of Liaoning Xianrendong. Axis 1 (RDA1) and axis 2 (RDA2) explain 92.03% of the cumulative relationship between physicochemical soil characteristics and stand species diversity. The first two axes respond to the gradient changes of four stand species diversity indices with 14 soil factors. The RDA (Fig 4) analysis showed that soil conductivity, pH, organic matter, P, Ca, and N were positively correlated with the Gleason abundance index (arbors), with soil P having the greatest influence. Soil conductivity, capacitance, K, and Fe were positively correlated with the Shannon–Wiener diversity index, Simpson dominance index, and Pielou’s evenness index, with soil K having the greatest influence. The four stand species diversity indices showed a positive and high degree of correlation with the Shannon–Wiener diversity index, Simpson dominance index, and Pielou’s evenness index. Overall, soil organic matter, Ca, N, and K, were significantly correlated with the stand species diversity index. Among them, soil Ca had the highest explanation and contribution. N ranked second, and K ranked third. Therefore, the species diversity of forest stands in Liaoning Xianrendong was mainly influenced by Ca, N, and P soil levels.

**Table 6:** Ranking of redundancy analysis between soil characteristics and species diversity in Layer A of a typical forest stand in Xianrendong, Liaoning Province.

Test Factor	Explanation rate (%)	Contribution rate (%)	F	*P*
Ca	42.0	45.6	20.4	0.002
N	16.9	18.4	12.0	0.002
K	10.2	11.1	10.1	0.006
P	8.6	9.4	2.4	0.138
Organic matter	4.5	4.9	5.4	0.026
Fe	2.4	2.6	3.1	0.058
Temperature	1.7	1.8	2.4	0.096
Mg	1.6	1.7	2.3	0.122
Capillary pore space	1.2	1.4	2.0	0.174
Compactness	1.2	1.3	1.8	0.190
Conductivity	0.7	0.7	1.1	0.312
Natural water content	0.5	0.5	0.7	0.438
pH	0.3	0.4	0.5	0.568
Bulk weight	0.2	0.2	0.3	0.692

The relationship between soil properties and stand species diversity is complex, and different soil properties lead to different vegetation types. The forest composition in this study area was dominated by arbor, and the species diversity index of arbor was as follows: Gleason abundance index was positively correlated with conductivity, pH, organic matter, Ca, N and P; Pielou’s evenness index with bulk weight and Fe. Gleason abundance index was negatively correlated with capillary pore space, bulk weight, drainage capacity, soil water storage and capillary water holding capacity; Simpson dominance index and Shannon-Wiener diversity index with capillary water holding capacity, drainage capacity and soil water storage; Pielou’s evenness index with Ca and N. The natural moisture content and clay particles are neutral feedback. The positive feedback of soil can lead to the formation of dominant species, which is not conducive to plant coexistence and the maintenance of system diversity, while the negative feedback of soil is conducive to species coexistence, community construction and the maintenance of species diversity. In this study area, the feedback mechanism is complex, which is conducive to maintaining the species diversity and stability of forest community.

## Discussion

Soil is the nutrient bank of plants, and plants are one of the important soil forming factors. The growth of plants affects the soil properties, and the change of soil properties will in turn affect the growth and health of the plants themselves and their coexisting plants. The process of such plants changing the soil properties first, and then the soil in turn affects the growth of plants, is called the "Plant-soil feedback" theory. Considering the theory of plant–soil feedback (PSF) [10], the zonal forest vegetation developed in certain climatic zones affects soil properties in various ways, such as plant root undergrowth, secretion, apoplast, precipitation trapping in the forest canopy, and microclimate regulation. It includes soil texture and structure, mineralization, and humification of soil organic matter, soil thermal properties and water circulation, soil biological community composition, etc. It is the dominant small-cycle biological process of material forming and evolving in the region’s soil. These changes in soil properties affect the growth and development of forest vegetation as well as the species composition, structure, and stability of forest communities. They are the sequestration base and nutrient reservoir for the growth and development of zonal forest vegetation in the region. This plant–soil feedback reflects overall changes in the soil’s abiotic and biotic factors due to forest vegetation at the temporal scale. It also reflects reciprocal positive, negative, or neutral feedback in the above-ground part of the forest ecosystem on a spatial scale.

The Liaoning Xianrendong National Nature Reserve belongs to the humid-temperate monsoon climate zone, which is influenced by the oceanic climate. It is located at the intersection of the two floras in North China and Changbai. The representative species of flora in North China mainly include *Pinus densiflora*, Quercus acutissima, *Quercus variabilis*, *Quercus dentata*, *Quercus aliena*, etc. The representative species of flora in Changbai mainly include *Quercus mongolica*, *Juglans mandshurica*, etc. These conditions have led to the development of nine typical zonal forest vegetation types with characteristics of both North China and Changbai flora. This region also contains brown forest soil and brown loam formed by the long-term combination of forest vegetation, climate, soil-forming parent materials, and topographic conditions. The Liaoning Xianrendong National Nature Reserve is an important site for material cycling and energy conversion between the biotic and abiotic environments, and its soil belongs to the core of the forest ecosystem. Forest soil is a multi-component complex of solid, liquid, and gas phases with complex physical, chemical, and biological properties. In addition to reflecting soil fertility, it coordinates the necessary growth and living conditions of nutrients, water, air, and heat for forest communities to grow, develop, reproduce, and spread. In this study, it was found that the soil compactness, total porosity, natural water content, organic matter, Ca, N and P of the community of *Pinus densiflora* + *Quercus acutissima*—*Parthenocissus tricuspidata* + *Zanthoxylum schinifoilum*—*Amphicarpaea edgeworthii* + *Cardamine leucantha* + *Arisaema angustatum* var. *peninsulae* community were higher, which was conducive to plant growth. It also reflects that the soil physical and chemical properties of the study area are suitable for the growth and development of the *Pinus densiflora* + *Quercus acutissima* mixed needle-broad and the maintenance of stand stability, which is consistent with the phenomenon that there is a large area of natural secondary *Pinus densiflora* + *Quercus* top communities in the study area. The above findings are similar to those of Song in the Daxingan Forest area [15], Cheng in the Simian Mountains of Chongqing [16], Zhu in the Qingshuihe County public welfare forest area of the Inner Mongolia Autonomous Region [17], Zema in the Castilla-Laman Nature Reserve in Eastern Spain [18], and Bay-ranvand in the forests along the plains of the Erburz Mountains in Northern Iran [19]. The soil fertility status and the water-holding and water-retention capacity of the mixed coniferous forest communities were superior to other forest stands.

The species diversity of zonal forest vegetation in Liaoning Xianrendong National Nature Reserve reflects the structural characteristics of the forest community and reveals mutual feedback among species and their relationship with the soil environment. In this study, the Gleason abundance index, Shannon–Wiener diversity index, Simpson dominance index, and Pielou’s evenness index of nine typical stands in the study area were (0.007±0.003) species/m^2^, 0.828±0.394, 0.458±0.216, and 0.639±0.279 for the arbor layer, respectively; (0.117±0.041) species/m^2^, 1.487±0.317, 0.695±0.103 and 0.835±0.060 for the shrub layer, respectively; (4.111±0.875) species/m^2^, 1.573±0.167, 0.726±0.042, and 0.838±0.061 for the herbaceous layer, respectively. Comparatively, the Gleason abundance index (arbors, shrubs), and Pielou’s evenness index for *Pinus densiflora* + *Quercus acutissima*—*Parthenocissus tricuspidata* + *Zanthoxylum schinifoilum*—*Amphicarpaea edgeworthii* + *Cardamine leucantha* + *Arisaema angustatum* var. *peninsulae* communities were higher, which coincided with the results of the soil properties evaluation and reflecting the positive feedback effect of the above-ground to below-ground components of the study area’s forest ecosystem. The above findings are similar to those of Varga [20] in Western Canada, where mixed forests are more conducive to plant coexistence and community diversity.

The Pearson’s correlation analysis showed that the correlation between soil nutrients and species diversity was complex, with positive feedback, negative feedback and neutral feedback, and the complex feedback relationship was conducive to maintaining the species diversity of the stand. Specifically, positive feedback was found in the soil’s physicochemical characteristics, including physical characteristics and bulk weight with the Pielou’s evenness index (arbors); drainage capacity with the Shannon–Wiener diversity index (herbaceous) and Simpson dominance index (herbaceous). chemical characteristics and conductivity with the Gleason abundance index (arbors, shrubs); pH with the Gleason abundance index (arbors), Simpson dominance index (shrubs) and Pielou’s evenness index (herbaceous); organic matter with the Gleason abundance index (arbors, shrubs) and Shannon–Wiener diversity index (shrubs), Simpson dominance index (shrubs) and Pielou’s evenness index (herbaceous); Ca with the Gleason abundance index (arbors, shrubs) and Shannon–Wiener diversity index (shrubs); Fe with Pielou’s evenness index (arbors); N with the Gleason abundance index (arbors, shrubs), Shannon–Wiener diversity index (shrubs), and Simpson dominance index (shrubs); P showed significant or obvious positive correlation with the Gleason abundance index (arbors, shrubs), Shannon–Wiener diversity index (shrubs), Simpson dominance index (shrubs) and Pielou’s evenness index (herbaceous), The results showed that there was a positive feedback between the above soil physicochemical characteristics and stand species diversity. The soil’s physical characteristics and temperature with the Shannon–Wiener diversity index (shrubs), Simpson dominance index (shrubs) and Pielou’s evenness index (shrubs); compactness with the Gleason abundance index (herbaceous); capillary pore space with the Gleason abundance index (arbors), Shannon–Wiener diversity index (shrubs), Simpson dominance index (shrubs); bulk weight with the Gleason abundance index (arbors, shrubs, herbaceous), Shannon–Wiener diversity index (shrubs, herbaceous), Simpson dominance index (shrubs, herbaceous) and Pielou’s evenness index (herbaceous); capillary water holding capacity with the Gleason abundance index (arbors, shrubs), Shannon–Wiener diversity index (arbors, shrubs), Simpson dominance index (arbors, shrubs) and Pielou’s evenness index (shrubs); drainage capacity with the Gleason abundance index (arbors, shrubs), Shannon–Wiener diversity index (arbors) and Simpson dominance index (arbors); soil water storage with the Gleason abundance index (arbors, shrubs), Shannon–Wiener diversity index (arbors), Simpson dominance index (arbors) and Pielou’s evenness index (herbaceous). chemical characteristics and Ca with the Gleason abundance index (herbaceous) and Pielou’s evenness index (arbors); K with the Pielou’s evenness index (herbaceous); P showed significant or obvious negative correlation with the Pielou’s evenness index (arbors), The results showed that there was a negative feedback between the above soil physicochemical characteristics and stand species diversity. There was no correlation between soil natural water content and clay particles, which indicated that there was a neutral feedback between the above soil physical and chemical characteristics and stand species diversity. The redundancy analysis results also indicated that variation in the species diversity of typical forest stands in Liaoning Xianrendong was mainly influenced by the soil’s Ca, N, and K content.

Based on plant-soil feedback (PSF) theory, the feedback mechanism between soil physicochemical properties and species diversity of typical forest communities in Changbai transitional zone of North China is complex, with positive feedback, negative feedback and neutral feedback. Under the action of various feedbacks, it is not only conducive to the growth and stability of forest community in the protected area. It also has reference value for the forest communities around the reserve and the natural or planted forests in the transition zone. One shortcoming of this study is that it only considers the physical and chemical properties of the soil, and does not consider the biological properties of the soil. We will refine this difference in future studies.

## Conclusions

1. Soil is the core of terrestrial ecosystem, with complex physical and chemical properties. It not only provides the necessary growth medium to support plants, but also provides the nutrient bank for plant growth. As an important soil forming factor, plants affect a variety of soil properties through root binding, secretions, litter and regulation of field microclimate, etc. The changes of these soil properties will in turn affect the growth and health of plants and their co-existing plants. This process of plant-soil interaction is called "plant ⁃ soil feedback".
  The mutual feed-effect of nine typical stand plant-soil in Xianrendong, Liaoning Province was represented by Gleason abundance index (arbors) with conductivity, pH, organic matter, Ca, N and P; Pielou’s evenness index (arbors) with bulk weight and Fe. Significant negative correlations between the Gleason abundance index (arbors) with capillary pore space, bulk weight, drainage capacity, soil water storage and capillary water holding capacity; Simpson dominance index and Shannon–Wiener diversity index with capillary water holding capacity, drainage capacity and soil water storage; Pielou’s evenness index (arbors) with Ca and N. The natural moisture content and clay particles are neutral feedback. Therefore, in Xianrendong National Nature Reserve of Liaoning Province, which is located at the intersection of North China and Changbai flora, the plant-soil feedback of typical zonal stands has positive, negative and neutral feedback effects in terms of species coexistence and stand species diversity. The complex feedback mechanism is conducive to the formation of forest community dominant species in the transition zone. It also maintained the species diversity and stability of forest communities in the transition zone. In view of the complexity of the mechanisms affecting soil characteristics and species diversity, further studies are needed.
2. This study investigated the relationship between soil traits and species diversity of typical stands in Xianrendong National Nature Reserve, Liaoning Province, which is of great significance for the effective maintenance of species diversity and community stability of natural stands in the transitional zone between Changbai flora and North China flora, and also provides a reference for the construction and management of artificial vegetation in Changbai flora and North China flora transition zone. Through plant-soil feedback directed regulation, reasonable management measures were selected for natural forest protection and plantation construction, and the advantages of forest community species diversity and soil physicochemical properties driving ecosystem versatility were fully utilized to realize the synergy of forest community and ecosystem services.

## Supporting information

S1 File (XLSX)

## References

[1] Zhu HJ, Chen JF, Chen SL, He YG. *Soil Geography*. Beijing: Higher Education Press; 2010.

[2] Appiah-Badu K, Anning AK, Eshun B, Mensah G. Land use effects on tree species diversity and soil properties of the Awudua Forest. *Global Ecology and Conservation*. 2022; 34: 1–13.

[3] Zhou RH, Su TC, Yu J, Xiang L, Chen CL, Zhang HW, et al. Species diversity and soil physicochemical properties of evergreen broad-leaved forest in Bifeng Gorge. *Journal of ecology*. 2012; 41(01): 1–8.

[4] Li MJ, He ZS, Jiang L, Gu XG, Jin MR, Chen B, et al. Altitudinal pattern and driving factors of species diversity and phylogenetic diversity in Daiyun Mountain. *Acta EcologicaSinica*. 2021; 41(3): 1148–1157.

[5] Onyekwelu JC, Mosandl R, Stimm B. Tree species diversity and soil status of primary and degraded tropical rainforest ecosystems in south-western Nigeria. *Journal of Tropical Forest Science*. 2008; 20(3): 193–204.

[6] Li X, Zhang XM, Wang YS, Han W, Yu LM. A study on the diversity of poisonous plants in Xianrendong National Nature Reserve, Liaoning. *Journal of Tonghua Normal University (Natural Science)*. 2013; 34(3): 46–52.

[7] Zhang XY, Wang YT, Tang LL, Li X, Zhang HQ, Wang YS, et al. Study on the diversity of medicinal vascular plants in Xianrendong National Nature Reserve, Liaoning Province. *Journal of Jilin Normal University (Natural Science Edition)*. 2013; 34(1): 97–99.

[8] Liu L, Huang GH, Zhang M, Yang RF. Analysis of seed flora and similarity with other mountainous areas in Xianrendong, Liaoning. *Northeast Normal University Newspaper (Natural Science Edition)*. 2014; 46(4): 116–121.

[9] Zhang L, Sun XY, Li SY, Zhang JD, Zhao JY, Fan JG. Soil carbon and nitrogen distribution characteristics of typical forest stand forests in Xianrendong, Liaoning Province. *Journal of Jilin Agricultural University*. 2017; 39(2): 183–188.

[10] Wang GZ, Jia JY, Zhang JL. Advances in plant-soil feedback theory and its application to natural and farmland ecosystems. *Acta ecologicasinica*. 2021; 41(23): 9130–9143.

[11] Wan DM, Sui GL. *Comprehensive Scientific investigation report of Xianrendong National Nature Reserve*, *Liaoning Province*. Liaoning University Press, China; 2022.

[12] Forestry Administration of the People’s Republic of China. *Forest soil analysis method (Forestry Industry Standard of the People’s Republic of China)*. China Standards Press China; 1999.

[13] Yuan ZY. Species diversity and niche characteristics of forest communities in Xianrendong, Liaoning Province. M.Sc.Thesis, Dalian: Liaoning Normal University. 2021.

[14] Cao L. Study on basic soil characteristics and heavy metal pollution in the lower reaches of Biliu River. M.Sc.Thesis, Liaoning Normal University. 2018.

[15] Song QL, Dong XB. Comprehensive evaluation of the stability of communities of different types of low-quality forests in Daxing’anling Mountains. *Forestry Sciences*. 2014; 50(6): 10–17.

[16] Chen JH, Zhang HJ, Wang W, Du SC, Li GP, Ren G. Evaluation of soil conservation function of five plantations in Siawan Mountain, Chongqing. *Journal of Beijing Forestry University*. 2009; 31(6): 54–59.

[17] Zhu JZ, Qin FC, Li L, Yang ZQ, Fang F, Zhao Q. Comprehensive evaluation of soil fertility of typical stand of artificial forest in loess hilly region. *Soil and fertilizer in China*. 2022; (2): 9–16.

[18] Zema DA, Stan J, Plaza‐Alvarez PA, Xu X, Stan J, Carra BG, et al. Effects of stand composition and soil properties on water repellency and hydraulic conductivity in mediterranean forests. *Ecohydrology*. 2021; 14(3): 1–13. doi: 10.1002/eco.2276

[19] Bayranvand M, Akbarinia M, Jouzani GS, Gharechahi J, Alberti G. Dynamics of humus forms and soil characteristics along a forest altitudinal gradient in hyrcanian forest. *IForest—Biogeosciences and Forestry*. 2021; 14. doi: 10.3832/ifor3444-013

[20] Varga P, Chen HYH, Klinka K, Gharechahi J, Alberti G. Tree-size diversity between single and mixed-species stands in three forest types in western Canada. *Canadian Journal of Forest Research*. 2005; 35(3): 593–601.

